# Methods of Honey Bee Stinger Removal: A Systematic Review of the Literature

**DOI:** 10.7759/cureus.8078

**Published:** 2020-05-12

**Authors:** J. Austin Lee, Eunice Singletary, Nathan Charlton

**Affiliations:** 1 Emergency Medicine, University of Virginia, Charlottesville, USA

**Keywords:** honey bee, stinger, envenomation, first aid, wilderness medicine, emergency medicine, hymenoptera

## Abstract

Honey bee envenomations are a common occurrence and cause localized morbidity but rarely cause systemic symptoms or death in humans. Honey bee stingers have a uniquely designed venom sac with a piston-containing bifurcated stinger that can remain in human skin and continue injecting venom after stinging. For some time, it has been proposed that a retained honey bee stinger should be scraped out by a dull edge, as opposed to pinching and pulling out the stinger, in order to minimize the volume of venom injected. We undertook a literature review to evaluate the evidence regarding the effectiveness and safety of methods of honey bee stinger removal. The initial search identified 23 articles of interest; following title and abstract screening, two studies met the inclusion criteria. The included articles used different methods and models to evaluate the relationship between venom injection over time, and one of these studies also compared different methods of stinger removal. The literature review was limited by the small number of studies on the topic, but both included studies include findings relevant to the clinical question of interest. Based on the available evidence, a retained honey bee stinger should be removed as quickly as possible, and there appears to be no disadvantage in doing it by pinching and pulling.

## Introduction and background

There are seven species of honey bees (order Hymenoptera, genus Apis) that exist worldwide; they are essential for the pollination of plants, including many food crops, and may also be used for harvesting honey [1]. They are found in large colonies and will sting to defend themselves or their nests; such stings are a common cause of morbidity in many regions of the world [1]. Honey bee venom contains several active compounds that lead to pain and cellular injury, including the proteins melittin (a hemolytic factor, and approximately 50% of the venom dry weight) and apamin [1]. The enzyme phospholipase A_2_ is believed to be the most allergenic and immunogenic protein in honey bee venom, and it may trigger anaphylaxis in hypersensitive individuals [1].

Honey bee envenomation can result in mortality in rare cases due to both allergic anaphylaxis and massive systemic envenomation [1]. It is unknown how many honey bee stings occur each year; however, the majority of victims only experience local symptoms, which include a raised erythematous area (wheal) that lasts for about 20 minutes accompanied by pain, itching, and swelling [1]. More severe and longer-lasting reactions often occur among those who have previously been stung by a honey bee [1].

It only takes one sting in a sensitized individual to produce anaphylaxis, which may result in death [2]. Anaphylaxis from Hymenoptera stings occurs in approximately 0.4-3.0% of the United States population and is the leading cause of death from animal venom with 40-50 deaths reported per year [3]. In patients with anaphylactic reactions, respiratory tract obstruction is the leading cause of death, followed by cardiovascular collapse [4]. Most deaths from honey bee stings occur among hypersensitive individuals who are only stung once, most often in the head or neck, and are usually aged more than 40 years [1]. Death can also occur from large volume envenomation; it is estimated that 500-1,500 honey bee stings are needed to produce a fatal systemic envenomation, and it is more likely to occur with more aggressive Africanized honey bees, which are a hybrid and invasive species in the Americas [4].

Unlike some other Hymenoptera species, a honey bee can only sting a victim once due to its single barbed stinger that is designed to detach from the bee; the stinger remains in the target tissue with the barbed end inhibiting removal once the sting occurs [1]. In a honey bee, the venom apparatus consists of a proximal venom sac and a distal bifurcated stinger with a piston-like mechanism that, even after detachment from the bee, functions independently to continue to pump venom into the wound and further imbed the stinger into the victim [5]. Analysis of the bee stinger has shown that a muscular movement, via a piston-like injection mechanism, results in venom flowing into the wound, and as such, it would seem that the method of removal may not be as important as the rapidity of removal [1,5].

Traditional first-aid recommendations are based on the assumption that venom can be squeezed from this venom sac during removal, which would actually increase the volume of venom injection and therefore worsen the envenomation. Hence, it has been recommended that the sac should not be pinched or squeezed during the removal of the venom-stinger apparatus from the skin. Prior recommendations, including those of the American Red Cross, advise removing the retained venom apparatus by scraping it out [6]. This is done as close to the base of the embedded stinger at the skin surface as possible, using the edge of a dull object (such as a credit card) to avoid squeezing the venom sac [6].

The objective of this systematic literature review was to determine, following honey bee envenomation in human adults and children, what is the most appropriate and effective method for removal of a retained honey bee stinging apparatus in the skin with regards to outcomes of localized reaction, pain, anaphylaxis, and need for further care.

## Review

Review methods

A systematic review was conducted according to the Cochrane Handbook for Systematic Reviews of Interventions (http://handbook-5-1.cochrane.org), and results are reported according to the Preferred Reporting Items for Systematic Reviews and Meta-Analysis (PRISMA) guidelines [7]. As this is a review and not a research study, no institutional review board approval was sought for this work.

Search strategy, information sources, and eligibility criteria

With the help of a medical librarian, a search strategy was developed including the terms “sting removal” or “stinger removal,” “bee” (or “bees” or “honey bee” or “Hymenoptera”), and “sting” or “stinger.” The following databases were included in the search with no date restrictions: PubMed, OVID - EBM Reviews, Cochrane DSR, ACP Journal Club, DARE, and Google Scholar. A further manual search was conducted based on the bibliographies of the articles discovered in the initial search. The search was limited to English language-only sources. Due to the anticipated low number of studies, both human and animal research was considered for inclusion. The search was initially performed on June 28, 2017, with a repeat search conducted on April 5, 2020, to identify any new articles published after the original search. Of note, this review excluded any research into or works regarding ocular honey bee stings since it is a rare occurrence with a unique set of potential complications and management concerns.

Risk of bias and certainty of available evidence

The risk of bias was assessed using the Cochrane risk of bias tools, including the SYstematic Review Centre for Laboratory animal Experimentation (SYRCLE) and the Risk Of Bias In Non-randomized Studies of Interventions (ROBINS-I). The certainty of evidence across outcomes was assessed through the Grading of Recommendations, Assessment, Development and Evaluation (GRADE) methodology [8]. The details regarding these bias and certainty scoring tools can be found in the Appendix.

Study selection

**Figure 1:**
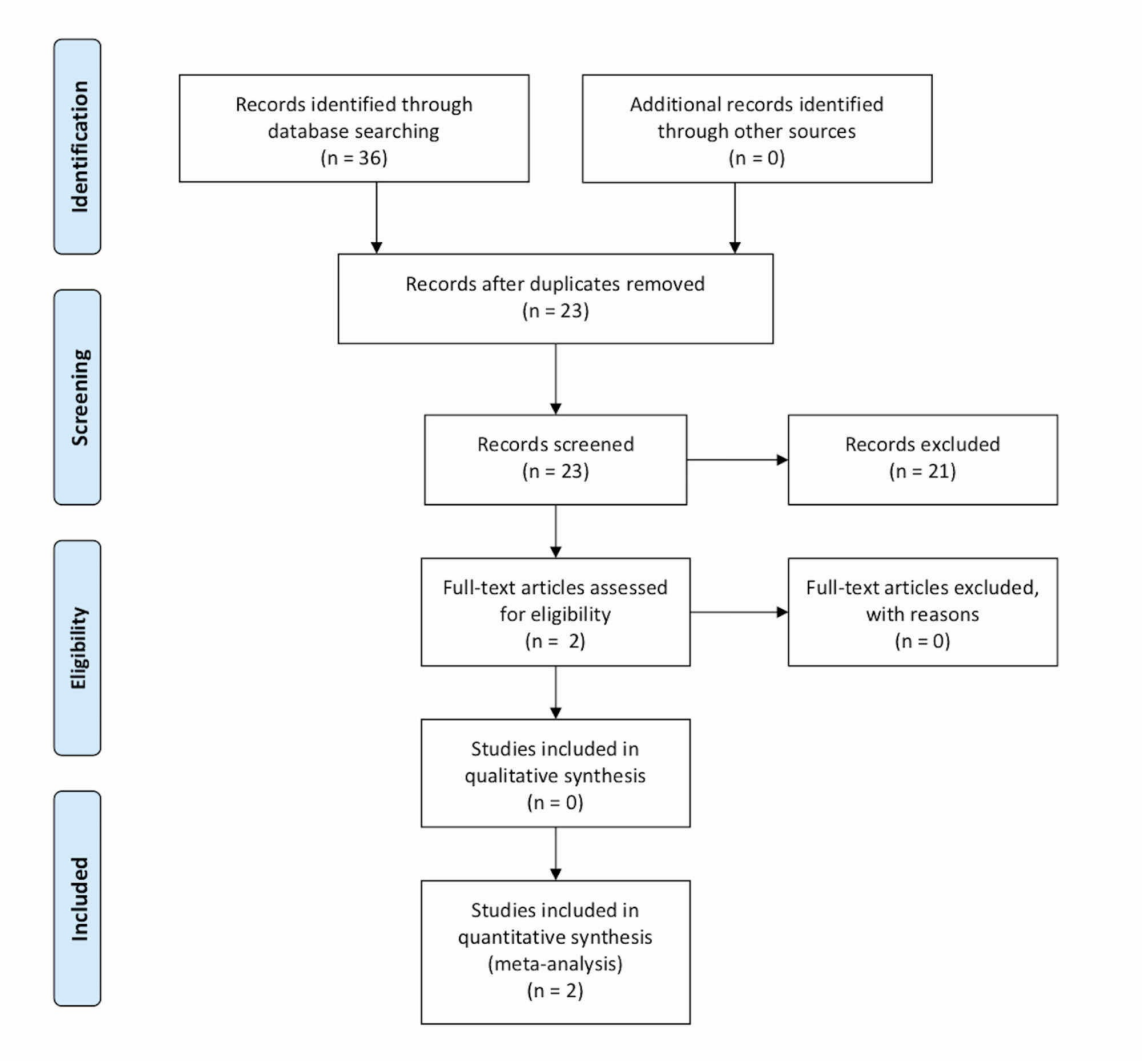
Literature review flow diagram

Two reviewers (NC and ES) independently reviewed titles and abstracts to determine eligibility for inclusion and, after a consensus was reached, the included studies were reviewed for quality of evidence and interventions and outcomes. A total of 23 studies were identified by the initial search strategy after all duplicates were removed. No additional studies were identified by the manual search. After the title and abstract screening, two studies were evaluated for full-text review, and both were included for analysis. A flow diagram that charts the review process is shown in Figure 1.

Study characteristics

Given the small number of studies identified for inclusion, no data were extracted for pooled-analysis or meta-analysis.

Review of results

The two studies identified for inclusion utilized different methodologies and models to evaluate the depth, volume, and duration of honey bee stinger envenomation [9,10]. The design, outcomes, key findings, biases, and uncertainty of these studies are summarized in Table 1.

**Table 1:** Characteristics, methods, and findings of included studies

Article	Study design	Outcome measured	Key findings	Bias	Certainty of evidence
Schumacher et al. [9]	Observational trial using rabbit and paper models	Evaluated depth of stinger insertion and volume of envenomation (as seen by protein assay and pre-post weight measurements)	In a rabbit model, the depth of stinger advance was correlated to longer time in the skin; the protein assayed for residual venom in the stinger apparatus was found to be inversely related to time in a paper model. It was seen that there was a statistically significant association between venom delivery (measured by weight) and time in both rabbit and paper models; venom delivery appeared to be maximally complete within 30 seconds	High/serious - SYRCLE assessment	Very low
Visscher et al. [10]	Quasi-experimental study in humans	Contrasting removal methods (scrape vs. pluck) as well as varying time of envenomation and subsequent measured wheal size	Found a statistically significant increase in wheal area with increasing time to stinger removal wheal area was approximately a log-linear function of dose. No statistically significant difference in wheal size per method of removal, though wheals were on average smaller when pulled as opposed to scraped (74 vs 80 mm^2^). Some stingers broke off in the skin with the scraping method versus no breakage using the pulling method	Serious - ROBINS-I	Very low

In the Schumacher study, a rabbit model was utilized to measure the depth of stinger advance over time [9]. These authors also measured the residual venom in the stinger apparatus at different time points after rabbit envenomation in order to best estimate the total volume of envenomation [9]. In a similar paper-model study design, the weight of venom ejected from the stinger was weighed at different time points [9]. Interestingly, both models in these observational trials found that the venom delivery appeared to be completed by 30 seconds [9]. In this study, the authors did not directly evaluate pain, anaphylaxis, or the need for further treatment after honey bee envenomation.

Visscher et al. used human subjects in a randomized controlled series of experiments and found that the size of localized reaction (wheal) increased with time of envenomation [10]. This study found no statistically significant difference between the methods of stinger removal (grasping-pulling compared with scraping) for the outcome of wheal size, though the size of the local reaction was, on average, smaller (74 mm^2^) when the stinger was pulled when compared with scraping for removal (80 mm^2^) [10]. Although not initially a part of their research question, it is interesting to note that Visscher et al. found that, in their experiments, several stingers broke off and were retained in the skin when using the scraping method of removal as opposed to the grasping-pulling method [10]. In this study, while the authors evaluated the means of stinger removal and the relationship with a localized reaction, they did not directly address or study outcomes of participant pain, anaphylaxis, or the need for further care.

Findings from both studies suggest that stinger removal should occur within the first few seconds as Visscher demonstrated a significant increase in wheal size over the first eight seconds, and Schumacher noted that envenomation appears to be exhausted by 30 seconds [9,10].

Risk of bias within studies and across studies

Overall, the certainty of the evidence was very low. This was primarily due to the studies being observational in nature and downgraded for risk of bias, imprecision, and indirectness. Both of the included studies used structured methodologies to attempt to limit the bias in their work. In the observational work by Schumacher et al., the use of standardized rabbit and paper models limited selection bias but neither of these is equivalent to humans [9]. The authors also engage in selective reporting with incomplete outcome data. The use of a melittin assay as a surrogate for envenomation does raise the risk of detection bias in this section of their study. By and large, this study did not clearly elucidate many of the variables that can lead to bias, and as such, the study has a high risk of bias overall.

In the work by Visscher et al., a quasi-experimental methodology was employed to limit bias [10]. The use of the surrogate of wheal size for the volume of envenomation does also introduce some bias in the authors’ ability to detect the outcomes of concern. There does not appear to be any clear performance bias, nor attrition or reporting biases; however, the risk of confounders is rather high in this study and there are serious risk-of-bias concerns.

Neither study included for review was funded by industry and neither appears to have any other systematic biases. To the best of our knowledge, there is no clear systematic publication bias or selective reporting occurring among researchers looking at honey bee envenomation in humans.

Summary measures and synthesis of results

Unfortunately, because of the limitations caused by the heterogeneity and paucity of available literature directly answering our research questions, we were unable to calculate any summary measures or extrapolate any meta-analysis of the available literature.

Summary of evidence

While the breadth of data is limited, the biomechanical design of the barbed piston mechanism of the honey bee stinger and the best available evidence suggest that removal of a honey bee stinger as quickly as possible may be of greater importance than the actual technique of stinger removal. The available research indicates that the rapidity of stinger apparatus removal, rather than the method, is what dictates the amount of venom deposited [9,10]. Rapid stinger removal has the potential to limit the size of the local reaction, theoretically limiting pain and other potential first-aid treatments that may be needed.

The Visscher study suggests that the method of removal (grasping and pulling versus scraping it out) is not important when removing the stinger. Yet, there was some suggestion in this same study that grasping and pulling the stinger apparatus rather than trying to scrape it out results in a lower rate of stinger breakage, thereby resulting in a lower rate of the retained foreign body [10].

Although Schumacher et al. did not look at the specific methods of stinger removal, it would seem that any removal technique that minimizes time with the stinger in the skin will decrease the localized reaction, pain, the chance of anaphylaxis, and need for further treatment or care [9]. In many real-world instances, pulling the stinger out will likely be quicker than finding a suitable thin dull object for scrapping a honey bee stinger off. For these reasons, it seems that the preferable method of stinger removal is grasping and pulling the stinger out. Given limits in the available data, it does not appear unsafe to scrape out the stinger. No data is available to determine whether or not the rapid removal of the stinger would mitigate the risk of anaphylactic reaction in a sensitized individual.

Furthermore, our study’s initial questions regarding the removal method and the incidence of anaphylaxis, or the need for further treatment and care, have not been studied or published in the literature. Similarly, studies in children have not addressed these same questions.

Limitations

Despite the large burden of human morbidity from honey bee stings, the identifiable differences in outcome measures among varied stinger removal techniques have limited the impetus for further investigation. Subsequently, our work is limited by the small number of studies investigating this subject. Additionally, the ability to accurately quantify the volume of envenomation can be difficult, even in a controlled research environment. Furthermore, both of the studies included in our review have their own shortcomings and limitations. The Schumacher study is limited by its use of non-human models (rabbits and paper), which makes the findings difficult to apply to honey bee envenomation in humans. The Visscher study was limited in size and also relied heavily on their use of wheal size as an imprecise indicator of envenomation volume. Both studies have significant risk-of-bias concerns; the Schumacher study did not report on a number of factors affecting bias and reported incomplete data, while the Visscher study includes significant confounders [9,10]. Lastly, no identified literature is available on honey bee envenomation in children though differences in the child, adolescent, and adult physiology may impact both localized and systemic reactions.

Future research

The findings of our systematic review give rise to several other clinical questions that are potential areas for future study. These include whether the rapidity of removal can ameliorate systemic allergic reactions, and if certain methods of stinger removal reduce or increase the breakage of the stinger or retention of foreign body and the need for future care. Furthermore, efforts to look at the differences between young children, adolescents, and adults, and local and systemic reactions to honey bee envenomation would be of scientific and clinical value.

## Conclusions

Available published research on the method and speed of honey bee stinger removal is limited. However, despite recommendations that bee stingers should be removed with a scraping dull-edge approach, the best available evidence, as presented here, suggests that after a honey bee sting, a residual embedded stinger should be removed as quickly as possible. Given the time required to find a dull-edged scraping device along with the evidence that grasping the stinger apparatus does not induce greater volume of envenomation, it would seem that it is advantageous to quickly remove the stinger by whatever means possible; and this most often will be by grasping and pulling out a retained honey bee stinger.

## Appendices

**Table 2:** SYstematic Review Centre for Laboratory animal Experimentation (SYRCLE), risk of bias for Schumacher et al.

Study	Schumacher et al. [9]
Year	1994
Design	Observational
Industry funding	None
Sequence generation	Unclear
Baseline characteristics	Unclear
Allocation concealment	No
Random housing	Unclear
Blinding of caregivers	Unclear
Random outcome assessment	Unclear
Blinding of outcome assessor	Unclear
Incomplete data	Yes
Selective outcome reporting	Yes
Overall risk of bias	High/serious

**Table 3:** Risk Of Bias In Non-randomized Studies of Interventions (ROBINS-I), risk of bias for Visscher et al.

Study	Visscher et al. [10]
Year	1996
Design	Observational
Total patients	1
Population	Stings to the forearm of one patient multiple times with treatments randomized
Confounding	Serious
Selection of participants	Low
Classification of interventions	Low
Deviation from intended intervention	Low
Missing data	Low
Measurement of outcomes	Low
Selection of reported result	Low
Overall risk of bias	Serious

**Table 4:** Grading of Recommendations Assessment, Development and Evaluation (GRADE) for the studies meeting inclusion criteria

Certainty assessment							Certainty
Study	Study design	Risk of bias	Inconsistency	Indirectness	Imprecision	Other considerations	
Schumacher et al. [9]	Animal Study	Serious	Not serious	Serious	Serious	None	Very Low
Visscher et al. [10]	Observational	Serious	Not serious	Not serious	Serious	Only one researcher stung multiple times	Very Low
